# Identification of *MEF2B* and *TRHDE* Gene Polymorphisms Related to Growth Traits in a New Ujumqin Sheep Population

**DOI:** 10.1371/journal.pone.0159504

**Published:** 2016-07-29

**Authors:** Li Zhang, Xiaomeng Ma, Junli Xuan, Huihua Wang, Zehu Yuan, Mingming Wu, Ruizao Liu, Caiye Zhu, Caihong Wei, Fuping Zhao, Lixin Du

**Affiliations:** Institute of Animal Sciences, Chinese Academy of Agricultural Sciences, Beijing, 100193, China; China Agricultrual University, CHINA

## Abstract

2 SNPs were discovered in our previous genome-wide association study (GWAS): s58995.1 (rs420767326 A>G) in *MEF2B* gene and OAR3_115712045.1 (rs401775061 A>C) in *TRHDE* gene, which were significantly associated with post-weaning gain in sheep. Herein, we performed a replication experiment to investigate single nucleotide polymorphisms (SNPs) within the *MEF2B* and *TRHDE* gene exons, the 5′untranslated regions (within 1000bp), the 3′ untranslated regions (within 1000bp) and their associations with Ujumqin sheep growth traits in 4-month age and 6-month age, respectively. Finally,3 SNPs were selected to be investigated including 1 SNP in 3′untranslated regions in *MEF2B* gene (rs417014745 A>G) and 2 SNPs in *TRHDE* gene (rs426980328 T>C and rs430810656 G>A).The χ2 test showed all the 3 variations were in Hardy–Weinberg equilibrium (*P*>0.05) status. Association analysis suggested that rs426980328 T>C was significantly associated with body weight and chest girth in 4-month age (*P*<0.05). rs430810656 G>A exhibited extremely significant association with body weight and chest girth in 4-month age (*P*<0.01). rs417014745 A>G was extremely significantly associated with body weight and chest girth in 4-month age and chest girth in 6-month age (*P*<0.01), and it was also significantly associated with body weight in 6-month age (*P*<0.05). Combined effect analysis indicated significant associations between the combinations of rs426980328-rs417014745, rs430810656-rs417014745 and several growth traits (*P*<0.05). These results suggested *MEF2B* and *TRHDE* genes affected growth traits in Ujumqin sheep and the combination effect of the two genes also played a significant effective role. These SNPs might have potential value as genetic markers for growth traits and it could be used in Ujumqin sheep breeding in future. Further studies are necessary to confirm our findings.

## Introduction

Growth traits are significant economic traits in sheep, advances of molecular genetics have opened appealing perspectives for the identification of functional genes through genomics technology, SNPs have been widely used for QTL detection and localization for complex traits in many species[1]. Abundant SNPs have been discovered by using modern sequencing technologies and bioinformatics tools, which allowed us to better understand the association between genomic variations and different traits. Our previous GWAS results showed that 2 SNPs: s58995.1(rs420767326 A>G) in *MEF2B* gene (Genbank XM_015095748) and OAR3_115712045.1 (rs401775061 A>C) in *TRHDE* gene (Genbank XM_004007369) were significantly associated with post-weaning gain in sheep [2]. *MEF2B* gene is one of the myocyte enhancer factor-2 gene family members (*MEF2A*, *MEF2B*, *MEF2C* and *MEF2D*) in vertebrates, which play a significant role in the regulation of muscular growth and development. *MEF2B* gene had a lower homology in different species, and it was also distinct from the rest gene family members. It was located on chromosome 5 and widely expressed in various organizations [3, 4], and it is crucial to cell development, embryonic development, the differentiation and regeneration of muscle tissue, the differentiation of nervous system and liver fibrosis. Expression of MEF2B mRNA and myocyte fibroblast proteins were higher in spindle cells than that in cobblestone cells. Knockdown of *MEF2B* in a cobblestone cell line abolished EGF-induced upregulation of MEF2, including vimentin and non-muscle caldesmon proteins, yet enhanced basal expression of mesenchymal vimentin and fibronectin[5].*MEF2B* mutations could be linked to CREBBP and EP300 mutations, and to recurrent Y641 mutations in EZH2 [6]. Study revealed MEF2B-Nox1 signaling was critical for stretch-induced phenotypic modulation of vascular smooth muscle cells [7]. In mouse, *MEF2B* was co-expressed with *MEF2C* throughout the early stages of cardiogenesis, it was up-regulated in *MEF2C* mutant mouse, consistent with the possibility that it may partially substitute for *MEF2C*[8].*MEF2B* also had an impact on the expression of *MEF2* target genes owing to the reduced acetylation of nucleosomes near these genes. As a novel candidate gene for growth traits detected by GWAS, however, no study was found on associations between *TRHDE* gene and sheep growth traits.*TRHDE* is thyrotropin-releasing hormone degrading enzyme which encodes a member of the peptidase M1 family. It is reported *TRHDE* gene was associated with neuroglioma in human[9], OAR3_115712045.1 identified by our GWAS analysis was located within a QTL which was found to affect internal fat amount in Merino sheep[10]. *TRHDE* protein was an extracellular peptidase that specifically cleaved and inactivated the neuropeptide thyrotropin-releasing hormone, which regulated appetite and metabolism [11, 12]. Knockdown of *TRHDE* gene in drosophila sensory neurons resulted in altered impaired nociception and cellular morphology[13]. *TRHDE* gene in goat was identified by the multiple genomic signatures of selection study, the results showed it could directly or indirectly influence traits for adaptation to hot arid environments[14].Based on these previous studies, further study on *MEF2B* and *TRHDE* genes were taken to investigate the relationship between SNPs within them and Ujumqin sheep growth traits, respectively, as well as their combined effect on Ujumqin sheep growth traits, in order to validate the GWAS results and screen casual genetic variants as genetic markers that benefit for the sheep growth performance in an independent sheep population.

## Experimental Section

### Ethic statement

The methods of this study were conducted in accordance with the Guidelines for Experimental Animals established by the Ministry of Science and Technology (Beijing, China).All experimental protocols were approved by the Science Research Department (in charge of animal welfare) of the Institute of Animal Sciences, Chinese Academy of Agricultural Sciences (CAAS) (Beijing, China).

### Phenotypic data collection and DNA extraction

Totally of 343 Ujumqin sheep were selected as experimental population, including 218 samples (111 rams and 107 ewes) collected at Dong Ujumqin Banner (Inner Mongolia Autonomous Region, China, N45° 30′, E116° 57′)and the remaining 125 samples (54 rams and 71 ewes) were from Zhenglan Banner (Inner Mongolia Autonomous Region, China, N42°14′,E115°30′). The feeding was in line with the Instructive Notions with Respect to Caring for Laboratory Animals that was published in 2006 by the Science and Technology Department of China (Approval No. S20072911). We collected 343 blood samples and each was 25 mL in 4-month age. We also measured the growth traits including body weight, body height, body length, chest girth, shin circumference in 4-month age and body weight, body height, body length, chest girth, shin circumference, chest width and chest depth in 6-month age. 343 genomic DNA were isolated from blood samples using a TIANamp Genomic DNA kit (TianGen, Beijing, China) according to the manufacturer’s instructions finally. In order to identify potential SNPs, a DNA pool (50 ng/uL /sheep) was constructed using 30 blood samples and primers were designed with Primer3 web based Program (v.0.4.0.) (http://bioinfo.ut.ee/primer3-0.4.0/), to amplify all the exons and 1000 bp of 5’-UTR and 3’-UTR based on the reference sequence. The Primer pairs used for scanning SNPs were in Table 1.

**Table 1:** Primer pairs used for scanning SNPs within sheep *MEF2B* and *TRHDE* gene.

PRIMER	Primer sequence(5'-3')	Annealing Temperature(°C)	Product (bp)	Extend region
TRH-2F	TGGCGAAATATGCATGACGT	59.3	595	Exon 2
TRH-2R	CCTCTCCGACTCTGAAACCA			
TRH-3F	CAGCGTGCTTTGATATATGAACA	59	300	Exon3
TRH-3R	TCCTTCCAACACAATAAGCAAGA			
TRH-4F	ACTGTGTTTGTTAAAGCCTTTGG	60.3	226	Exon4
TRH-4R	ACTTACACCTGATCCTTTTCTCA			
TRH-5F	GAGGTGATTTTATCTGATGCCCA	59.5	232	Exon5
TRH-5R	GAGGTGATTTTATCTGATGCCCA			
TRH-6F	ACCTGTGATGTTGTTCCACC	59.6	229	Exon6
TRH-6R	AGAATTTGGCCAGGAGAGTGA			
TRH-7F	TGCCAGAACCAGGTTTGAA	60.6	683	Exon7
TRH-7R	TCGATTTCCCAAGCTGTGAA			
TRH-8F	ACTGGAAATTGCTTAGCCTTTGA	58.8	249	Exon8
TRH-8R	GCATATCAAGAAAAGCGAAACGT			
TRH-9F	TGTTGATGCCTTGCCTAGTT	58.3	840	Exon9
TRH-9R	ACAACTTTGGTACGCTGTCAC			
TRH-10F	CTCCTCACATTTATGACTGGGG	59.2	266	Exon10
TRH-10R	TGAGACCAAGTAGTTCCTCAGAG			
TRH-11F	CTCTCTTACCCCTAAAATGCTGT	59.8	533	Exon11-12
TRH-11R	TTGATCAAGCTTCTCCTGGC			
TRH-13F	GCCTCATCATGTATCTTTTCTGC	60.3	350	Exon13
TRH-13R	TGCCATTAGTGTCATGCAGA			
TRH-14F	TCAGTTGTCAGAGCAAAGTGA	59	824	Exon14-15
TRH-14R	TCTCGTCTTGTTTCTCTCCAGT			
TRH-16F	ACTGTACTAAGGCAGCATTTGTC	59.8	999	Exon16-17
TRH-16R	TCTGGATCTTGCTTCTACCACA			
TRH-18F	CACAGCCTTGAACAGCGTTA	59	295	Exon18
TRH-18R	ACTGGAAAGAAATGGTTGAAGTC			
TRH-19F	TGTGCTGTCTGTTTACCATTCT	60	247	Exon19
TRH-19R	TTCCTCATTCCACAGCAGGT			
ME-5'-1F	TTGGTTTGTTTGGTTCTGGTTT	57.9	545	5'-UTR 500bp
ME-5'-1R	CAGCAGGGCACAGTGATCT			
ME-5'-2F	AGTGTCTGCTGTGTGGATGA	58.2	600	5'-UTR500-1000bp
ME-5'-2R	TGGTCCAGAATGCGTGAGAT			
MEF2B-1F	TTCCATCCACCTGCTCTGTT	60.1	234	Exon1
MEF2B-1R	CCAGGCTGTGTTTCTTCTGC			
MEF2B-2F	CCTCTCACCACCCTGAACTT	58.8	335	Exon2
MEF2B-2R	AACCGCAACGCTCATCTTTT			
MEF2B-3F	CTGTCTCCCCTTCCCACAG	59.9	242	Exon3
MEF2B-3R	AGTCCTTCTCTCAGCCTCAG			
MEF2B-5F	CCGCTCCTCTCTCTCTCTTT	61	360	Exon5
MEF2B-5R	GTCAGAACCGGAGCCATTTC			
ME-3'-1F	TCCCTTACCCTTTGCTCCTG	58.6	568	3'-UTR500bp
ME-3'-1R	CAGCATTCACTCACGGATCC			
ME-3'-2F	GCTGGGCTATTTACCCTCCA	60.6	664	3'-UTR500-1000bp
ME-3'-2R	TACAGAGGGTTGCTTGGAGG			

### SNP detection and genotyping

The pooled DNA were amplified with a programmable thermal cycler, (Bio-Rad, Thermal Circlers, Mexico).The PCR reaction mixture consisted of 25 μL, containing 2 μL pooled DNA, 1 μL of 5′ primers, 1 μL of 3′ primers, 12.5 μL 2× Taq PCR MasterMix and 8.5 μL double-distilled water. The PCR reaction protocol was 5 min at 94°C for initial denaturing followed by 35 cycles at 94°C for 30 s; Tm for 30 s; 72°C for 30 s; a final extension at 72°C for 10 min. The PCR products were stored at 4°C. Then PCR products were detected via 2% agarose gel and visualizing under UV rays to confirm the amplification. The desired product were sequenced by Tianyi-Huiyuan Gene Sequencing Co.Ltd (Beijing, China).Then the results of the sequencing were aligned with standard sequence and the SNPs were detected using DNAMAM 5.2.10 software (Lynnon BioSoft, Quebec, ONT, Canada) and Chromas 2 software. Considering the compatibility of the primers used to extend the SNPs genotyped in the next MALDI-TOF assay process, 3 SNPs were then genotyped in 343 Ujumqin sheep using MALDI-TOF assay (Mass ARRAY; Sequenom Inc, San Diego, CA, US).

### Statistical analysis

Microsoft Excel 2013 (Microsoft Inc, Redmond, WA, USA) was used to calculate the allele frequencies, polymorphic information content (PIC), heterozygosity(*He*), and homozygosity (*Ho*). SPSS 22.0 software (SPSS Inc., Chicago, IL, USA) was used to analyze the associations between the SNPs and growth traits of the sheep. The Hardy–Weinberg equilibrium were tested for each site through the chi-square test. The following model was used to check the single gene effect on the sheep growth traits:

$$\text{Yi }=\text{μ}+\text{ Gi }+{\text{p}}_{\text{i}}+{\text{m}}_{\text{i}}+{\text{e}}_{\text{i}},$$

where Yi was the trait measured in individual, μ was the overall mean, Gi was a fixed effect corresponding to the genotype of polymorphisms, p_i_ was the gender effect, m_i_ was the farm effect, and e_i_ was a random residual effect. The model of association analysis between combined genotypes and growth traits was as follow:

$$\text{Yi }=\text{μ}+{\text{G}}_{\text{i}}+{\text{p}}_{\text{i}}+{\text{m}}_{\text{i}}+{\text{e}}_{\text{i}}$$

Y_i_ was the trait measured in individual, μ was the overall mean, G_i_ was the combined genotype effect of the 2 genes, p_i_ was the gender effect, m_i_ was the farm effect, and e_i_ was a random residual effect.

Significant differences among genotypes and their corresponding mean growth trait values were calculated using Duncan’s multiple-range test. *P*<0.05 was considered significant. *P*<0.01 was considered extremely significant. Numbers less than 6 of the combined genotypes were not included in the association analysis.

## Results

### SNP detecting and genotyping

DNA pool sequencing showed totally 2 SNPs were identified in *MEF2B* gene and 5 SNPs in the *TRHDE* gene, considering the compatibility of the primers used to extend the single site in the next MALDI-TOF assay process, rs417014745 A>G in the 3’-UTR of *MEF2B* gene and 2 SNPs in the exon2 (rs426980328 T>C), exon14 (rs430810656 G>A) respectively were selected to be genotyped in a new Ujumqin sheep validation group including 343 samples. All the 3 SNPs were classed into 3 genotypes in the experimental population.

### Genetic parameters calculation

Genetic parameters were analyzed in the experimental population (Table 2), the results showed all the sites were in Hardy-Weinberg equilibrium status (*P*>0.05). rs426980328 and rs417014745 were in low polymorphic information content status (PIC<0.25), while rs430810656 was in moderate polymorphic information content status (0.25<PIC<0.5).The homozygosity of the 3 sites were higher than the heterozygosity.

**Table 2:** Genetic parameters of the SNPs in Ujunmqin experiment population

Locus	Genotype	Genotypic Frequency	Allele	Allelic frequency	He	Ho	PIC	H-W test (P value)
rs426980328	CC(291)	0.86	C	0.93	0.14	0.86	0.13	0.7845
	CT(44)	0.13	T	0.07				
	TT(2)	0.01						
rs430810656	AA(172)	0.51	G	0.27	0.39	0.61	0.31	0.0826
	GA(144)	0.43	A	0.73				
	GG(18)	0.06						
rs41701474	GG(242)	0.72	G	0.84	0.27	0.73	0.24	0.5152
	GA(85)	0.25	A	0.16				
	AA(9)	0.03						

### Association of single nucleotide polymorphism with growth traits in sheep

The results of single nucleotide polymorphism association analysis revealed the rs417014745 that located in 3’-UTR of *MEF2B* gene was extremely significantly associated with body weight, chest girth in 4-month age (*P*<0.01) and chest girth in 6-month age (*P*<0.01), it was also significantly associated with body weight in 6-month age (*P*<0.05).On rs417014745, sheep carrying AA genotype were significantly better than the GG or GA carriers on body weight and chest girth in 4-month age. As to the growth traits in 6-month age, sheep with AA genotype performed extremely significantly better than the GG or GA genotype carriers on body weight, the allele A might be the superior allele on rs417014745.

rs426980328 that located on exon2 of *TRHDE* gene was significantly associated with body weight, chest girth in 4-month age (*P*<0.05). On rs426980328, sheep with TT genotype were significantly greater than those CC or CT genotype carriers on body weight and chest girth in 4-month age, which could be observed with overall 3.91kg heavier and 3.21cm bigger from phenotype, respectively. Individuals with homozygous genotype TT on rs426980328 showed better growth performance on body weight, body length and chest girth in 6-month age than that with CT or CC genotypes. With regards to rs430810656, it was extremely significantly associated with body weight and chest girth in 4-month age (*P*<0.01). On rs430810656, individuals with AA genotype were overall 3.85 kg heavier in 4-month age and 3.70cm bigger in 4-month age chest girth than those with GG genotype. Sheep carrying AA genotype were overall 2.38kg heavier in 6-month age than those with GG genotype, individuals with homozygous genotype AA had higher body height, body length, chest girth and chest width than that with GG genotype in 6-month age as well. The association analysis results were in Tables 3 and 4.

**Table 3:** Associations of single marker of the genes with 4-month age growth traits. Data marked with the lower letters within the same rank mean significant difference (*P*<0.05), different uppercase letters indicate extremely significant difference (*P*<0.01).

Locus	Genotype	4-month age growth traits (Mean ± S.D.)				
		Body weight(kg)	Body height(cm)	Body length(cm)	Chest girth (cm)	Shin circumference(cm)
rs426980328	CC	28.59±5.45^b^	59.13+2.54	61.65±2.39	76.29±6.06^b^	7.73±0.45
	CT	30.20±5.24^b^	59.77+2.21	62.14±3.04	77.40±5.45^b^	7.87±0.55
	TT	32.50±4.24^a^	62.00+2.60	61.75±2.40	79.50±5.04^a^	8.00±0.11
	P	0.026	0.221	0.494	0.045	0.331
rs4308106565	AA	29.59±5.12^A^	59.26±.2.49	61.92±2.29	77.31+5.72^A^	7.76±0.48
	GG	25.74±5.70^B^	59.02±2.74	60.97±2.13	73.61±6.16^C^	7.75±0.42
	GA	28.14±5.52^C^	59.14±2.43	61.53±2.70	75.69±6.08^B^	7.72±0.46
	P	<0.001	0.846	0.1585	<0.0001	0.7100
rs417014745	AA	31.61±5.01^A^	60.55±2.69	62.66±2.44	79.33±4.45^A^	7.82±0.50
	GG	28.52±5.43^B^	59.12±2.30	61.57±2.50	76.08±6.10^B^	7.72±0.46
	GA	29.29±5.39^B^	59.30±2.91	61.91±2.08	77.15±5.64^B^	7.78±0.47
	P	0.003	0.218	0.051	0.005	0.057

**Table 4:** Associations of single marker of the genes with 6-month age growth traits. Data marked with the lower letters within the same rank mean significant difference (*P*<0.05), different uppercase letters indicate extremely significant difference (*P*<0.01).

		6-month age growth traits (Mean ± S.D.)						
Locus	Genotype	Body weight(kg)	Body height(cm)	Body length(cm)	Chest girth (cm)	Shin circumference (cm)	Chest width(cm)	Chest depth(cm)
rs426980328	CC	32.43±5.67	60.14±3.22	64.16±4.01	81.65±6.33	7.56±0.43	14.89±2.54	31.20±4.15
	CT	33.06±5.42	60.28±3.25	64.82±3.25	81.55±6.80	7.61±0.44	15.21±3.29	31.47±2.69
	TT	36.00±4.94	60.00±1.41	66.00±1.41	83.50±3.53	7.80±0.28	15.00±2.22	30.50±2.07
	P	0.171	0.950	0.179	0.460	0.539	0.805	0.972
rs430810656	AA	33.22±5.39	60.36±3.2	64.63±4.01	82.68±5.81	7.59±0.41	15.20±2.18	31.42±2.72
	GG	29.84±4.86	58.80±3.62	61.93±3.61	77.80±6.60	7.48±0.41	13.60±2.09	29.80±2.83
	GA	31.95±5.82	60.05±3.16	64.04±3.70	80.83±6.73	7.53±0.45	14.79±3.12	31.14±5.16
	P	0.907	0.911	0.551	0.810	0.753	0.794	0.933
rs417014745	AA	36.26±4.52^a^	61.25±2.76	65.00±4.00	85.25±7.59^A^	7.75±0.23	15.5.±2.56	30.37±2.44
	GG	32.41±5.73^b^	60.09±3.28	64.21±4.11	81.27±6.45^B^	7.54±0.45	15.03±2.87	31.07±2.82
	GA	32.50±5.35^b^	60.15±3.02	64.42±3.36	82.35±5.96^B^	7.62±0.38	14.57±1.83	31.78±2.43
	P	0.037	0.698	0.568	0.001	0.058	0.311	0.369

### Association of combined markers with growth traits in sheep

When combined the SNPs in *MEF2B* and *TRHDE* genes respectively, highly significant interactions were observed, the combination of the rs426980328 in *TRHDE* gene and rs417014745 in *MEF2B* gene showed significant association with body weightand chest girth in 4-month age (*P*<0.05), and chest girth in 6-month age(*P*<0.05). Sheep with the combined genotype CCAA had higher body weight, body length, chest girth in 4-month age and higher body weight, chest girth in 6-month age than the individuals with the other 4 combined genotypes. CCAA was the preponderant genotype. The combination of rs430810656 and rs417014745 exhibited significantly association with body weight and chest girth in 4-month age (*P*<0.05), the rs430810656-rs417014745 combined genotypes were also significantly associated with chest girth in 6-month age (*P*<0.05), GGAA was the preponderant combined genotype. The association analysis results were in Tables 5 and 6.

**Table 5:** Combined genotype effect analysis of rs426980328- rs417014745 and rs430810656- rs417014745 with 4-month age growth traits. Data marked with the lower letters within the same rank mean significant difference (*P*<0.05), different uppercase letters indicate extremely significant difference (*P*<0.01).

	4-month age growth traits (Mean ± S.D.)					
Loci	Genotype	Body weight(kg)	Body height(cm)	Body length(cm)	Chest girth (cm)	Shin circumference(cm)
rs426980328- rs417014745	CCAA	32.18±5.02^A^	60.75±2.81	62.62±2.61	79.87±4.43^a^	8.00±0.26
	CCGA	28.75±5.60^C^	59.17±3.01	61.94±2.20	76.74±5.93^bc^	7.75±0.50
	CTGA	31.55±2.21^AB^	59.05±1.77	61.72±1.56	79.05±3.04^ab^	8.00±0.25
	CCGG	28.16±5.31^C^	58.96±2.31	61.50±2.46	75.83±6.13^c^	7.70±0.44
	CTGG	29.93±5.81^BC^	59.98±2.33	62.26±3.37	77.04±5.96^bc^	7.83±0.61
	P	0.009	0.195	0.364	0.034	0.483
rs430810656- rs417014745	GAAA	29.85±5.31^a^	59.32±3.21	62.10±2.10	78.04±5.66^a^	7.77±0.54
	GAGA	28.83±5.16^ab^	59.12+2.20	61.68±2.06	76.37±5.61^ab^	7.80±0.40
	GAGG	26.68±5.71^bc^	58.85±2.79	61.35±1.37	74.35±4.24^bc^	7.78±0.48
	GGAA	29.51±5.11^a^	59.22±2.24	61.86±2.36	77.04±5.81^ab^	7.75±0.47
	GGGA	27.60±5.48^ab^	58.99±2.35	61.30±2.63	75.21±6.19^abc^	7.68±0.47
	GGGG	25.14±5.80^c^	59.13±2.84	60.72±2.53	73.16±7.29^c^	7.72±0.14
	P	0.025	0.613	0.502	0.027	0.496

**Table 6:** Combined genotype effect analysis of rs426980328- rs417014745 and rs430810656- rs417014745 with 6-month age growth traits. Data marked with the lower letters within the same rank mean significant difference (*P*<0.05), different uppercase letters indicate extremely significant difference (*P*<0.01).

	6-month age growth traits(Mean ±S.D.)							
Loci	Genotype	Body weight(kg)	Body height(cm)	Body length(cm)	Chest girth (cm)	Shin circumference(cm)	Chest width(cm)	Chest depth (cm)
rs426980328- rs417014745	CCAA	36.98±4.36	61.85±2.34	64.71±4.23	86.14±7.73^a^	7.78±0.22	15.85±2.54	30.42±2.63
	CCGA	31.94±5.32	60.17±2.98	64.20±3.29	82.20±6.13^b^	7.62±0.38	14.43±1.92	31.78±6.63
	CCGG	32.37±5.73	60.06±3.32	64.08±4.21	81.23±6.29^b^	7.52±0.45	15.02±2.73	30.99±2.79
	CTGA	34.62±3.53	60.00±3.54	64.75±3.10	82.50±5.34^b^	7.48±0.31	15.50±0.92	31.62±1.50
	CTGG	32.70±5.89	60.48±3.22	64.77±3.37	81.37±7.32^b^	7.65±0.47	15.20±3.73	31.48±2.99
	P	0.127	0.558	0.886	0.010	0.055	0.365	0.622
rs430810656- rs417014745	GAAA	32.81±5.05	60.19±2.80	64.50±2.99	83.63±6.11^A^	7.63±0.36	14.69±1.93	31.58±2.51
	GGAA	33.29±5.55	60.40±3.37	64.58±4.34	82.44±5.72^AB^	7.57±0.43	15.42±2.23	31.46±3.04
	GAGA	32.23±5.35	60.37±3.14	64.46±3.42	81.09±5.72^AB^	7.60±0.37	14.46±1.60	32.28±3.09
	GAGG	31.62±5.94	59.87±3.15	63.95±3.77	80.35±6.82^B^	7.50±0.48	14.81±3.51	30.71±2.77
	GGGA	30.42±5.65	58.28±2.92	62.57±3.50	80.57±5.31^B^	7.50±0.41	14.28±2.49	30.00±2.00
	GGGG	29.33±4.38	59.25±4.30	61.37±3.85	75.37±6.96^C^	7.46±0.45	13.00±1.60	29.62±3.54
	P	0.309	0.840	0.380	0.008	0.490	0.439	0.830

## Discussion

To validate the effect of promising SNPs identified by our previous genome-wide association study, we successfully genotyped 3 SNPs in a new validation group of 343 Ujumqin sheep with growth traits measured at 4-month age and 6-month age respectively. It showed all the 3 sites were in Hardy-Weinberg equilibrium (*P*>0.05) status, this might be owe to the closed environment where the Ujumqin sheep lived, which indicated that the sheep were not be extensively artificially selected. According to the standard of polymorphic information content, rs426980328 and rs417014745 were in low polymorphic information content status (PIC<0.25) and the heterozygosity of the 2 sites were also very low, indicating the genetic variation of these sites were not high, this might give rise to the experimental population were not highly selected, prompting that we could strengthen the artificial selection. While, the rs430810656 site was in moderate polymorphic information content status (0.25<PIC<0.5) and the genetic variation of rs430810656 was high, indicating that we could obtain more selection effect.

No research was found before we carried a relationship analysis between variations in *MEF2B*, *TRHDE* genes and growth traits in Ujumqin sheep. The 3 SNPs were proved to be significantly associated with several growth traits in a new population, implying these SNPs might be linked to a QTL which had an effect on different stages of sheep growth and development. *MEF2B* was involved in the transcription process of muscle specific growth factors and the cell differentiation of skeletal muscle and myocardium development. However, its genetic structure has not been widely investigated and the relationship with sheep growth traits remains unknown. Previous study revealed that the *MEF2B* gene was significantly associated with the meat trait and reproduction traits in pigs[15]. Genes preferentially regulated by *MEF2B* were associated with hepatic fibrotic pathways, ovarian cancer signaling and human stem cell pluripotency[16].Study revealed expression level of *MEF2B* was significantly positively correlated with myocyte fiber diameter (*P*<0.05) and significantly negatively correlated with myocyte fiber density (*P*<0.01)[17]. *MEF2B* was considered to be a good candidate gene for growth traits as it was a transcription factors which possessed DNA binding activities, it could be directly bonded to the specific promoters and enhancers of genes related to the muscle development. Previous study revealed in the absence of any *MEF2* gene family members, genes related to the development of muscle showed partial transcriptional disruption [18]. In the study of muscle development mechanism, MRFs (myogenic regulatory factors) was considered as a core factor in the signal transduction net, in the process of muscle cell development, MRF must interact with other factors to play its effect, including the interaction with the *MEF2* gene family. MRF4 promoter contains abundant information of muscle specific gene expression, but its full expression required MEF2 binding sites, mutations in *MEF2B* gene might alter binding site thus influenced the muscle development[19].

The expression pattern of Mef2b mRNA indicates that MEF2B, like its paralogs, may contribute to development and maintenance of a variety of tissue types [20]. *MEF2B* and its gene family members play an important role in cell proliferation and differentiation, embryonic development, muscle production, neural system differentiation and liver fibrosis. Therefore, it is reasonable to suggest that *MEF2B* mutations had a critical role in affecting the development and phenotype.

In single-locus analysis, the results showed the substitution of A with G on rs417014745 site lead to a consequence that sheep with mutation genotype AA had higher body weight, chest girth in 4-month age and higher body weight, chest girth in 6-month age than that in the wild genotype GG carriers. The detected SNP in the *MEF2B* gene in our study was located in 3’–UTR, studies proved that mutations in the 3’–UTR may affect the appropriate expression of genes[21, 22], the 3’–UTR of mRNA could affect the rates of localization, translation and mRNA specific degradation[21], more importantly, miRNA could interact with the 3’–UTR of mRNA to negatively regulate the gene transcripts, which might cause a gene to be silent[23]. The importance of the variations in the 3’–UTR could not be ignored, it might either directly have an effect on the expression of the gene or be linked to other variation in critically important regions of the genes that regulate gene expression. The substitution of rs417014745 might take effect through this way, further research of the miRNA and rs417014745 are necessary. Herein the allele A might be useful to improve the growth performance of sheep.

While the GWAS identified s58995.1 (rs420767326 A>G) in *MEF2B* gene showed no polymorphism in this experiment, Mass array genotyping results indicated that all the Ujumqin sheep were with GG genotype on the GWAS identified SNP locus. This might be caused by the following reasons: some mutations are specific to certain groups, and they can not be found when they are validated by other groups [24] or the numbers of the Ujumqin sheep were not that rich.In order to detect the SNP locus, we could expand the numbers of experiment population next.

As a novel candidate gene for sheep growth traits, seldom research on *TRHDE* gene were performed in human and sheep until now. TRHDE enzyme was an extracellular peptidase, it was considered to have a critical role in inhibiting the release of the neuropeptide effectively. A Genome-wide scan in porcine revealed a SNP near the *TRHDE* gene was associated with ear size at genome significance level[25]. Our study revealed rs426980328 and rs430810656 were significantly associated with Ujumqin sheep growth traits. Both rs426980328 and rs430810656 sites were synonymous mutations, it is widely considered that synonymous mutations had no effect on gene function and animal phenotype because these mutations could not lead to the change of amino acid in the process of protein synthesis[26].However, recent reports proved synonymous mutations were usually under the evolutionary pressure, which affected the gene function by changing the shear mode and stability of mRNA as well as changing the characteristics of protein subunits [27, 28]. Because of purifying selection, synonymous mutations might be fixed with a higher probability than neutral ones during evolution. The effect of synonymous mutations on gene function and phenotype were controlled by various factors. Zuo reported a synonymous 93G>A mutation in the *MC4R* gene which was significantly associated with back fat thickness, sheep with mutation genotype AA was significantly higher than those with wild genotype GG [22].In our study, the rs426980328 synonymous mutation improved the body weight, chest girth in 4-month age and body weight, chest girth in 6-month age compared with the wild genotype carriers. Generally speaking, although one amino acid could be encoded by several codons, the organism had a certain preference for the choice of the codon or tRNA in the translation process. On the rs426980328 site, the usage of the codon(UAC) to encode tyrosine was 25.2%, while the mutation type(UAU) was 14.3%.On rs430810656 site, the usage of the condon (GCA) to encode the alanine was 13%,while the mutation type (GCG)was 5.3%. (http://www.kazusa.or.jp/codon/).The usage rate change might lead to a deceleration in the incorporation rate of the amino acid in protein peptide synthesis process, and finally affect the phenotype of the organisms. The results suggested that rs426980328 and rs430810656 may be functional polymorphisms which could affect Ujumqin sheep growth traits. To the *TRHDE* gene, according to the compatibility of the primers used for Mass array genotyping, we could not obtain the genotypes of the sheep on the GWAS identified SNP locus.

Growth traits were quantitative traits controlled by multiple genes, they were not only influenced by the effect of single polymorphic loci but also the combined effect of multiple mutation sites [29]. Previous study was reported in small tailed Han ewes carried BB/G+ genotype (BMPR-IB and BMP-15) showed more litter size than those with either mutation alone, which proved the effect of multi-marker combination on the economic traits [30].Based on our data, we found that individuals with CCAA combined genotype of rs426980328 and rs417014745 were overall 4.12kg heavier and 4.04cm bigger than the CCGG combined genotype carriers on body weight and chest girth in 4-month age, and overall 4.74kg heavier and 4.94cm bigger than the CCGG carriers on body weight and chest girth in 6-month age, respectively. Individuals with CCAA combined genotype performed better on body weight, chest girth in 4-month age and body weight, chest girth in 6-month age than those with either mutation CC or AA genotype alone, which indicated that combined genotype might play a more effective role in body weight and chest girth in sheep, the combined genotype CCAA was the superior genotype in the experimental population. When we analyzed the combined effect of rs430810656 and rs417014745 on growth traits in sheep, the results showed that the individuals with GAAA genotype were overall 4.71 kg heavier and 4.88cm bigger on chest girth in 4-month age than those with GGGG carriers. Sheep with GGAA genotype were overall 3.96kg heavier in 4-month age and 7.07cm bigger on chest girth in 6-month age than those with the GGGG carriers. Both GAAA and GGAA were advantageous genotypes affecting the sheep growth performance and homozygous genotypes, and they were easily to be selected and fixed in sheep production, which suggested that we can look the sites as genetic markers and strengthen the artificial selection in sheep breeding.

We particularly noted that most of the SNPs were significantly associated with growth traits in 4-month age, but were not remarkably associated with growth traits in 6-month age, this might give rise to the weaning time, the weaning time was always in 4-month age, during 3-month age to 4-month age was the growth peak of sheep the candidate genes might play a part together with the growth environment and feed nutrition to jointly influence the growth traits effectively in this crucial time.

## Conclusions

Our results provide evidence that the *MEF2B* and *TRHDE* genes might be better candidate genes for Ujumqin sheep growth traits. These SNPs within them have potential value for growth traits of Ujumqin sheep. rs426980328-rs417014745 and rs430810656-rs417014745 pyramiding provided a way to accelerate the sheep growth traits improvement.
